# Association of urinary chlorpyrifos, paraquat, and cyproconazole levels with the severity of fatty liver based on MRI

**DOI:** 10.1186/s12889-024-18129-1

**Published:** 2024-03-14

**Authors:** Peiqi Ma, Hongliang Gao, Ning Shen, Lei Zhang, Yang Zhang, Kai Zheng, Boqun Xu, Jian Qin, Jian He, Tao Xu, Yan Li, Jing Wu, Yushan Yuan, Bin Xue

**Affiliations:** 1 Medical imaging center, Fuyang People’s Hospital, 236000 Fuyang, China; 2 Department of General Surgery, The Affiliated Changzhou Second People’s Hospital of Nanjing Medical University, Nanjing Medical University, 213003 Changzhou, China; 3 Core Laboratory, Department of Clinical Laboratory Sir Run Run Hospital, Collaborative Innovation Center for Cancer Personalized Medicine, Nanjing Medical University, 211166 Nanjing, China; 4 China Exposomics Institute (CEI) Precision Medicine Co. Ltd, 200120 Shanghai, China; 5 Jiangsu Engineering Research Center of Stomatological Translational Medicine, Nanjing Medical University, 210029 Nanjing, China; 6 Department of Obstetrics and Gynecology, the Second Affiliated Hospital of Nanjing Medical University, 210011 Nanjing, China; 7 Department of Orthopaedics, Sir Run Run Hospital, Nanjing Medical University, 211100 Nanjing, China; 8 Department of Nuclear Medicine, Affiliated Hospital of Medical School, Nanjing Drum Tower Hospital, Nanjing University, 210029 Nanjing, China; 9 School of Clinical Medicine, Wannan Medical College, 241000 Wuhu, China

**Keywords:** Exposure, Chlorpyrifos, Paraquat, Cyproconazole, Fatty liver disease

## Abstract

**Background:**

The objective of this study was to detect the urinary levels of chlorpyrifos, paraquat, and cyproconazole in residents living in Fuyang City and to analyze the correlation between these urinary pesticides levels and the severity of fatty liver disease (FLD).

**Methods:**

All participants’ fat fraction (FF) values were recorded by MRI (Magnetic resonance imaging). First-morning urine samples were collected from 53 participants from Fuyang Peoples’Hospital. The levels of three urinary pesticides were measured using β-glucuronidase hydrolysis followed by a. The results were analyzed by using Pearson correlation analysis and binary logistic regression analysis to reveal the correlation between three urinary pesticides and the severity of fatty liver.

**Results:**

53 individuals were divided into 3 groups based on the results from MRI, with 20 cases in the normal control group, 16 cases in the mild fatty liver group, and 17 cases in the moderate and severe fatty liver group. Urinary chlorpyrifos level was increased along with the increase of the severity of fatty liver. Urinary paraquat level was significantly higher both in the low-grade fatty liver group and moderate & serve grade fatty liver group compared with the control group. No significant differences in urinary cyproconazole levels were observed among the three groups. Furthermore, urinary chlorpyrifos and paraquat levels were positively correlated with FF value. And chlorpyrifos was the risk factor that may be involved in the development of FLD and Receiver Operating Characteristic curve (ROC curve) analysis showed that chlorpyrifos and paraquat may serve as potential predictors of FLD.

**Conclusion:**

The present findings indicate urinary chlorpyrifos and paraquat were positively correlated with the severity of fatty liver. Moreover, urinary chlorpyrifos and paraquat have the potential to be considered as the predictors for development of FLD. Thus, this study may provide a new perspective from the environmental factors for the diagnosis, prevention, and treatment of FLD.

## Background

Environmental chemicals, such as pesticides, have become a growing concern for human health because several health issues common non-communication diseases, including obesity and type 2 diabetes are attributed to these pesticides [1, 2]. Humans are more and more exposed to pesticides through food intake, accidental or occupational exposure during farming, contacting livestock treated with insecticides, and contaminated drinking water [3]. Despite the excessive use of pesticides is reduced, over 4 billion pounds of pesticides have been used on crops annually worldwide [4]. In China, the overuse of pesticides and the toxic residues of pesticides left in water pose great health risks to families in certain areas [5, 6]. A huge body of evidence exists on the possible role of pesticide exposures in the elevated incidence of human diseases such as cancers [7], Alzheimer [8], Parkinson [9], asthma [10], bronchitis [11], infertility [12], birth defects [13], autism [14], diabetes [15], and obesity [16]. Thus, understanding the relationship between chronic exposure to pesticides and human diseases becomes necessary.

Fatty liver is generally divided into alcoholic fatty liver diseases, non-alcoholic fatty liver diseases and metabolic dysfunction-associated fatty liver disease [17–19]. And The incidence of FLD increased in recent years [20]. With the rapid development of medical imaging such as ultrasound, computed tomography, or MRI, the understanding of fatty infiltration of the liver is becoming more and more objective [21]. However, these imaging examinations still presented some disadvantages including operator-dependent and subjective [22], slight lack of accuracy and risks of radiation exposure [23]. Moreover, they also tend to be costly and time consuming. Beyond that, in the era of life-omics, huge amounts of multi-omics data have been generated and widely used in diagnosis of FLD [24] such as radiomics analysis [25], proteomics [26], glycomics [27], lipidomics [28], metabolomics [29]. However, characteristics of exposomes which could reflect the impact of environmental pollution have not been studied. Therefore, combining exposomes and medical imaging diagnosis would provide a potential noninvasive, time-saving, and relatively simple tool to diagnosis of FLD.

As the largest solid organ, the liver serves as a pivotal role in metabolism including lipid and glucose metabolism as well as detoxication in the human body [30]. It reported that insecticides are easily deposited in the liver and adipose tissue which led to further long-term exposure to insecticides [31]. Chlorpyrifos is one of the predominantly used pesticides to kill mosquitoes, borers, cotton aphids, and pink bollworms which were likely to damage crops like wheat, rice, and cotton [32]. However, the massive use of chlorpyrifos affects soil fertility, modifies soil microbial community structure, and poses potential health risks to non-target organisms. Chlorpyrifos mainly causes inhibition of acetylcholinesterase enzyme and damages the nervous system of humans as well as the immune system, endocrine disruption, and embryonic disorders [33–35]. It was also reported that chlorpyrifos inhibits diet-induced thermogenesis in brown adipose tissue and this important factor contributing to the development of obesity and insulin resistance [36]. Paraquat is a cheap and effective herbicide widely used worldwide to remove weeds in vegetables, fruits, and cultivated crop fields [37]. Like with chlorpyrifos, paraquat can also cause soil and water pollution, and pose serious harm to the environment and organisms. Accidental or intentional ingestion of paraquat led to multiorgan failure through spreading to the lung, liver, kidney, thyroid gland and muscle through blood circulation is responsible for the death of paraquat poisoning [38]. Cyproconazole, developed in 1987, is a kind of triazole fungicide with the effect of anti-bacteria and plant growth regulators. It is typically applied as foliar sprays to restrain diseases by inhibiting fungal steroid demethylation [39]. Due to stability and solubility in water, cyproconazole piles up in the water and then causes harm to the human body [40]. Maternal exposure to certain triazole antifungal medication causes congenital malformations, including skeletal malformations. It was reported that exposure to cyproconazole induces adipogenesis while repressing skeletal development based on whole-embryo transcriptomics [41]. However, there are no published reports about the relationship between the accumulation of these three commonly used pesticides and FLD progression. Thus, exploring the relationship with the progress of FLD by detecting the concentration of these three pesticides in the human body could provide new ideas for the treatment and diagnosis of FLD.

Fuyang, neighboring Henan Province and located in the northwest of the Anhui Province, is a city that mainly developed on the primary industry (agriculture, forestry, animal husbandry, and fishery). Therefore, it is unquestionable that pesticide was under extensive use and residents are easily subject to environmental exposure to pesticide residues. Thus, effective and precise detection of pesticide accumulations in the bodies of residents may also provide important suggestions for health management.

In this study, we detected three common pesticides (chlorpyrifos, paraquat, and cyproconazole) in the urine based on LC-MS. Therefore, the objective of the present study was to provide baseline information on the levels of chlorpyrifos, paraquat, and cyproconazole in urine samples from residents living in Fuyang City and to assess the correlation between the level of pesticides exposure and the severity of FLD. More importantly, this study will provide a new reference direction for the diagnosis, prevention, and treatment of FLD as well as providing suggestions for environmental protection policies.

## Methods

### Study design and participants

All procedures, including sampling and examination, were conducted in accordance with the principles outlined in the Declaration of Helsinki and its subsequent revisions. A total of 53 participants from Fuyang peoples’ hospital were analyzed. All participants’ FF values were recorded by MRI fat quantitative examination (FF < 5% represented the normal group, 5 ≤ FF ≤ 14% represented mild fatty liver, and FF ≥ 14% represented moderate and severe fatty liver) [42]. The urinary level of chlorpyrifos, paraquat, and cyproconazole was measured by a high-performance liquid chromatography-tandem mass spectrometry-based method.

### Inclusion and exclusion criteria

Participants who had been diagnosed with fatty liver disease by MRI fat quantitative examination were recruited and individuals were excluded if: (a) A history of alcohol consumption, hepatitis, cirrhosis, or liver cancer; (b) A history of abdominal surgery; (c) Have taken drugs that affect blood lipid and blood sugar in the past six months; (d) Participants who are with genetic metabolic diseases or cardiovascular diseases; (e) Participants who could not complete MRI because of claustrophobia; (f) Poor image quality.

### Imaging detection of FLD

Philips Ingenia CX 3.0T magnetic resonance scanner was adopted with mDIXON-Quant scanning sequence and 8-channel abdominal special coil. Patients were asked to inhale, exhale and hold their breath during scanning. The imaging parameters were as follows: repetition time msec/echo time msec, 5.8/1.01, the field of view(FOV): 400 mm×350 mm×210 mm, voxel: 2.5 mm x 2.5 mm x 6 mm, NSA: 1, layer number 70. FF was obtained after scanning. The region of interest (ROI) was plotted on the right anterior lobe, right posterior lobe, and left lobe of the liver at the level of portal vein display on the post-processing workstation, respectively. The average values of the three were taken to represent the final liver FF value, and the ROI area was set to 200 mm².

### Sample collection and processing

Morning urine samples (second portion) were collected using plastic Vacuette® Urine Collection Cups (Greiner Bio-One International AG, Austria). Urine samples were filtered through a membrane filter of 0.22 μm. Using acetic acid-sodium acetate buffer (0.5 M) to adjust the pH of respective mixtures to 5.4. β-glucuronidase/arylsulfase (10 µL), and vitamin C (5 mg) were added and incubated overnight at room temperature to complete the enzymatic hydrolysis. Urine samples after enzymatic hydrolysis were extracted by solid-phase extraction with an SPE column (C18 ENVIJI 0.25 g) and then eluted with 2mL methanol and dried with nitrogen. Finally, 100 µL methanol was re-dissolved as the analyte to be determined. 50 µL of the analyte was tested by transferring it to a liquid chromatography bottle with a micro syringe, which was especially used for the injection analysis. Evaluation of chlorpyrifos, paraquat, and cyproconazole levels in the urine of participants was performed using LC-MS. The 1 µg/mL standard working solutions of chlorpyrifos, paraquat, and cyproconazole were prepared with methanol as solvent. The final standard working solution was 100 pg/mL after continuous dilution of 10^4^ times. Take chlorpyrifos as an example, different concentrations of chlorpyrifos standard working solution were prepared. 50µL of each chlorpyrifos standard working solution was transferred to a liquid chromatography bottle with a micro syringe, which was specially used to inject chlorpyrifos standard samples. Urinary TCPy level was detected to reflect the level of chlorpyrifos. Urinary paraquat and cyproconazole level were directly detected.

### Statistical analysis

One-way analysis of variance (ANOVA) was used to analyze differences among the different diagnostic groups concerning demographic information, physical examination indicators, and the urinary level of three pesticides. Bonferroni multiple comparison tests were used in the posttest. The association between the urinary level of three pesticides and the development of FLD was analyzed by using Pearson correlation. Risk factors for the development of FLD were analyzed by binary logistic regression. Receiver operating characteristic (ROC) curves were used to assess the diagnostic strength of urinary pesticides. A difference with *P* < 0.05 was considered statistically significant. All data were analyzed with R version 4.0.2.

## Results

### Study design and FF value determination

**Fig. 1:**
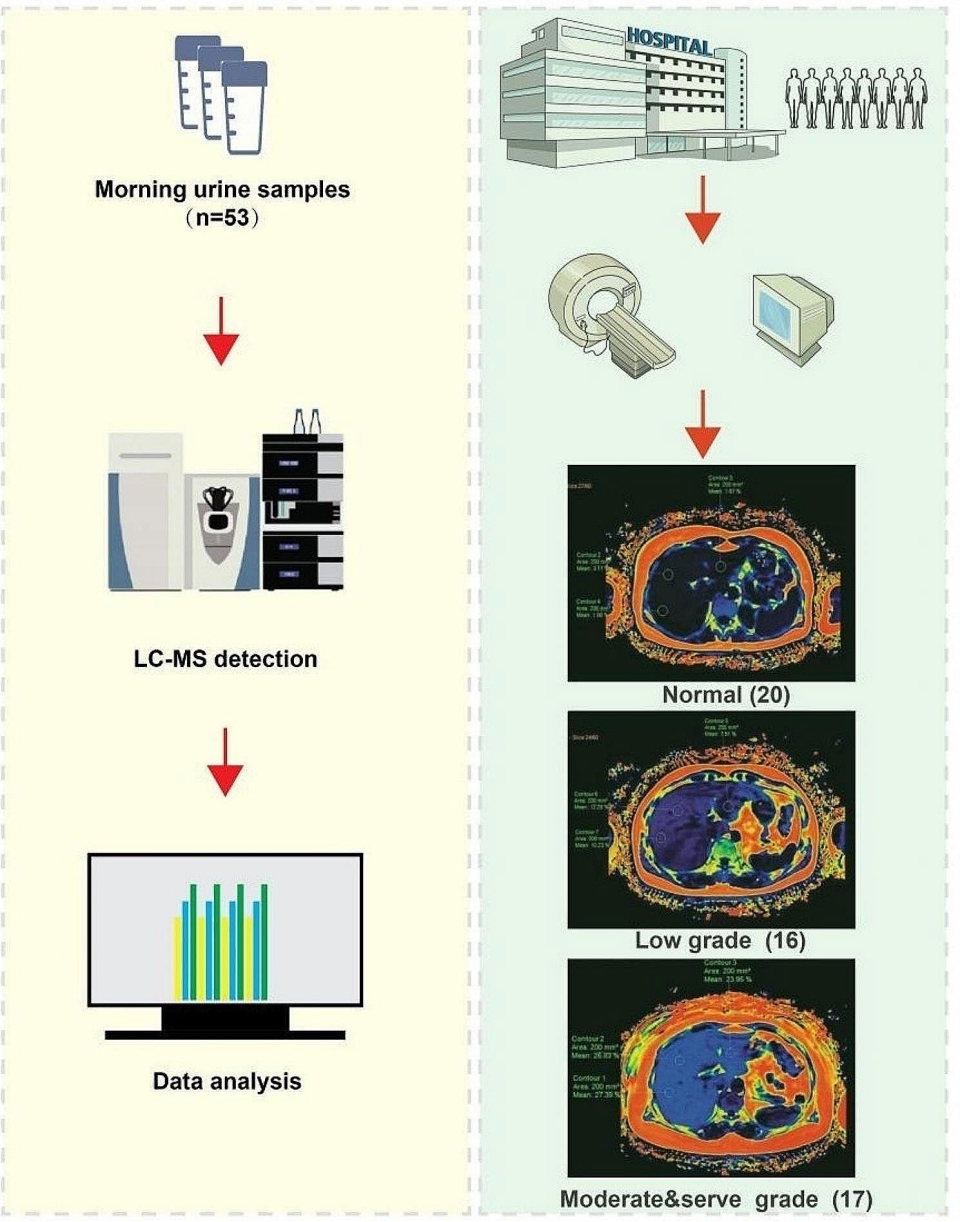
The schematic diagram of urinary pesticides detection and liver FF measurement

A total of 53 urine samples collected from enrolled individuals were detected by LC-MS method described. Besides, we divided them into 3 groups (control group, low-grade fatty liver group, and moderate&serve grade fatty liver group) according to the FF value (%) referring to the previous description, so there were 20 cases in the normal control group, 16 cases in the mild fatty liver group, and 17 cases in the moderate and severe fatty liver groups (Fig. 1).

### General data of enrolled individuals

The general data of enrolled individuals are demonstrated in Table 1. In total, 53 individuals including 31 males and 22 females were enrolled, aged 25–73 years, with an average age of 42.30 ± 12.05 years.

**Table 1:** Demographic characteristics of enrolled individuals

	Control (*n* = 20)	Low grade fatty liver (*n* = 16)	Moderate&Serve grade fatty liver (*n* = 17)	F value	P value
Male	9	10	13		
Female	11	6	4		
Age	43.80 ± 12.14	42.06 ± 13.18	40.76 ± 11.32	0.288	0.751
BMI	24.73 ± 3.33	28.13 ± 2.34	29.51 ± 1.51	17.178	< 0.001
Smoking	2/20	4/16	6/17		
Sedentary	7/20	12/16	14/17		
Urinary creatinine (mmol/L)	68.92 ± 20.16	68.56 ± 16.28	68.71 ± 11.21	0.002	0.998
TC (mmol/L))	4.94 ± 1.04	5.02 ± 0.67	5.21 ± 0.63	0.488	0.617
ALT(U/L)	22.00 ± 7.04	33.87 ± 15.17	40.06 ± 25.88	3.541	0.038
AST(U/L)	20.00 ± 3.00	20.87 ± 6.82	25.71 ± 8.66	3.194	0.051
GGT(U/L)	26.40 ± 20.90	41.18 ± 46.78	64.94 ± 50.97	4.156	0.021
ALP(U/L)	68.47 ± 19.95	74.18 ± 18.58	74.41 ± 30.76	0.380	0.686
TBA(µmol/L)	4.01 ± 2.61	4.30 ± 2.07	5.72 ± 4.33	1.509	0.231
TBIL(µmol/L)	11.08 ± 4.25	13.21 ± 7.28	11.79 ± 3.87	0.743	0.481
DBIL(µmol/L)	3.41 ± 1.44	3.81 ± 1.37	3.20 ± 0.88	0.959	0.390
IBIL(µmol/L)	7.88 ± 3.17	9.45 ± 6.14	8.54 ± 3.30	0.593	0.556

*Note* Statistical methods: One Way ANOVA. TC: Total cholesterol; ALT: Alanine aminotransferase; AST: Aspartate aminotransferase; GGT: Glutamyl transpeptidase; ALP: Alkaline phosphatase; TBA:total bile acid; TBIL:Total bilirubin; DBIL:Direct Bilirubin; IBIL: Indirect Bilirubin

### Urinary concentration of chlorpyrifos, paraquat, and cyproconazole

**Fig. 2:**
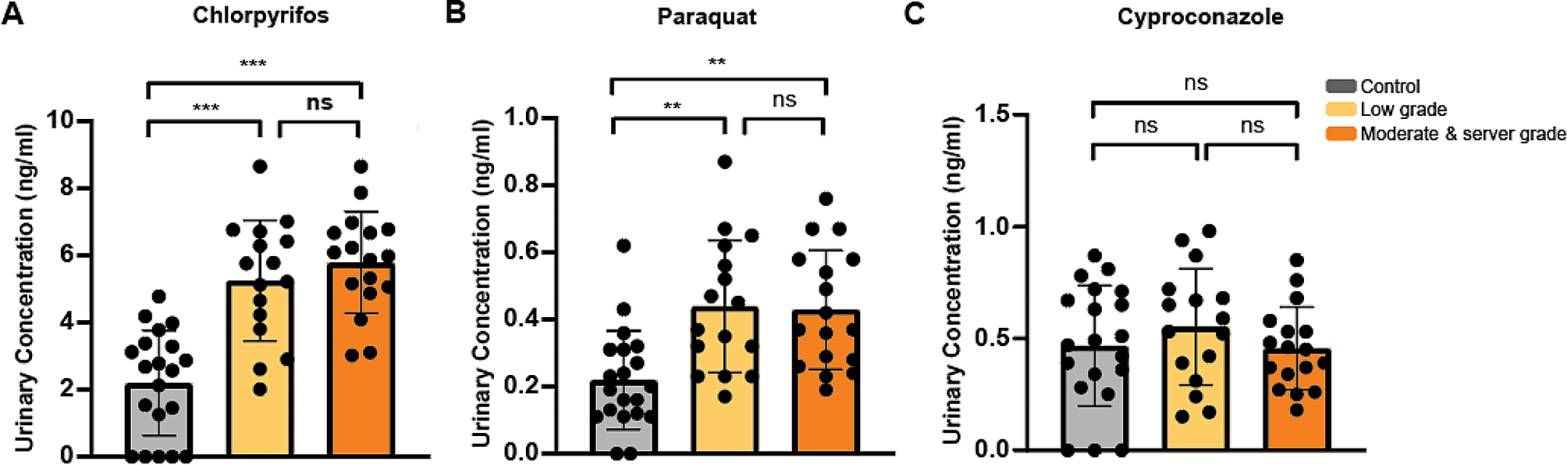
Urinary levels of chlorpyrifos, paraquat and cyproconazole. (**A**) Urinary concentration of chlorpyrifos; (**B**) Urinary concentration of paraquat; (**C**) Urinary concentration of cyproconazole

The urinary level of chlorpyrifos, paraquat, and cyproconazole was measured among different groups by a high-performance liquid chromatography-tandem mass spectrometry-based method. The results indicated that the urinary chlorpyrifos level increased as the severity of fatty liver increased (Fig. 2A). And the urinary paraquat level was significantly increased in the low-grade fatty liver group and the moderate & serve grade fatty liver group compared with the control group but there was no significant difference between the low-grade fatty liver group and the moderate & serve grade fatty liver group (Fig. 2B). In addition, no significant differences in urinary cyproconazole levels were observed among the three groups (Fig. 2C).

### Chlorpyrifos and paraquat were positively correlated with FF value

**Fig. 3:**
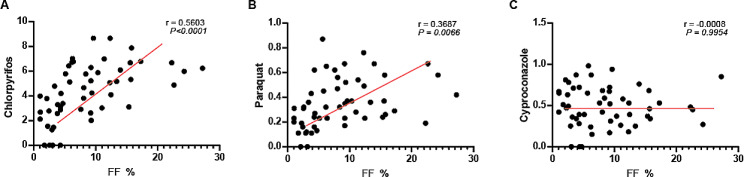
The association between three urinary pollutants levels and the severity of fatty liver. (**A**) The association between urinary chlorpyrifos level and FF%; (**B**) The association between urinary paraquat level and FF%; (**C**) The association between urinary cyproconazole level and FF%

To gain insights into the association between urinary chlorpyrifos, paraquat, and cyproconazole level and the severity of fatty liver, we performed a correlation analysis. We found that urinary chlorpyrifos and paraquat levels were significantly and positively correlated with FF value (*r* = 0.5603, *P* < 0.0001, *r* = 0.03667, *P* = 0.0066, respectively) (Fig. 3A&B). But urinary cyproconazole level showed no correlation with FF value (*r* = 0.0008, *P* = 0.9954) (Fig. 3C).

### Binary logistic regression analysis

Table 2 presents the risk factors for the development of FLD using binary logistic regression analysis. It was shown that chlorpyrifos was the risk factor that may be involved in the development of FLD. However, here it should be pointed out that even though paraquat could serve as a risk factor according to the regression analysis, we thought that this phenomenon was due to the significant degree of differentiation in urinary paraquat level. The results indicated that daily intakes and abnormal accumulation of chlorpyrifos may ultimately lead to FLD.

**Table 2:** Binary logistic regression analysis

	β	Wald	P value	OR (adjusted)*	95%C.I.	
					lower	Upper
Chlorpyrifos	1.732	10.96	0.001	5.65	2.027	15.75
Paraquat	9.209	9.816	0.002	9984.64	31.436	3171285.3
Cyproconazole	0.807	0.433	0.511	2.241	0.202	24.821

*Note* *Adjusted by age and gender

### Chlorpyrifos and paraquat could serve as independent predictors of FLD

**Fig. 4:**
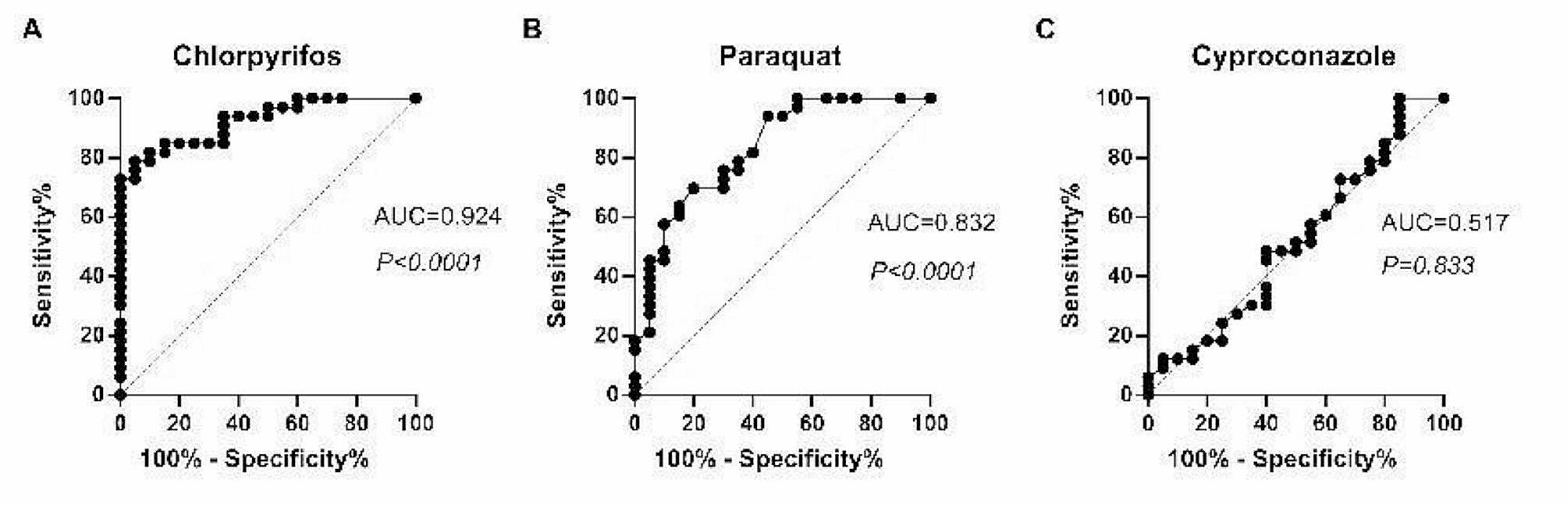
Receiver operating characteristic curve analysis of the three Eds. (**A**) A ROC curve and AUC values showing the discriminating capacities of chlorpyrifos; (**B**) A ROC curve and AUC values showing the discriminating capacities of paraquat; (**C**) A ROC curve and AUC values showing the discriminating capacities of cyproconazole

We further determined the sensitivity and specificity of urinary chlorpyrifos, paraquat, and cyproconazole levels for the diagnosis of FLD by receiver operating characteristic (ROC) curve analysis. The cut-off value was set at the point whose distance from the (sensitivity, specificity) = [1] reached the minimum. The AUC of chlorpyrifos, paraquat, and chlorpyrifos levels for FLD was 0.924,0.832 and 0.517 (Fig. 4) respectively which indicated that chlorpyrifos and paraquat may serve as potential predictors of FLD.

## Discussion

Long-term exposure to pesticides could cause dangerous effects on the skin, endocrine system, nervous system, and many other systems of the human body [43]. Therefore, regular monitoring of pesticide residues in human body can effectively prevent the occurrence of such diseases. Compared with serum and plasma, urine could better reflect the changes in human metabolism of pesticides because of the higher urinary metabolite concentration [44]. In our previous study, we had evaluated the urinary levels of dimethoate, bisphenol A, and benzo[a]pyrene and provided those baseline levels in healthy adults [45].

In this study, we evaluated the levels of 3 common-used pesticides in China and analyzed the correlation between these three pesticides with severity of FLD. we found that the urinary levels of chlorpyrifos and paraquat were higher in the people with FLD and were significantly elevated as the severity of FLD increased. As is well known, excessive fat deposition in hepatocytes, a hallmark of steatosis, is frequently associated with FLD [46, 47] and it was also reported that environmental pollutants tended to accumulate in liver and caused liver damage [31]. Therefore, this explains the abnormal higher levels of chlorpyrifos and paraquat in the people with FLD. Besides, these results coincided with the results from our previous study [45] that the accumulation level of environmental pollutants was higher in people with high BMI compared with people with low BMI.

Excessive accumulation of pesticides in hepatocytes certainly could affect basal hepatocyte functions and might contribute to the development of FLD. Therefore, we analyze the relationships between FLD and accumulation level of pesticides. And we found urinary chlorpyrifos and paraquat levels were significantly and positively correlated with severity of FLD. Additionally, although we did not find a significant correlation between urinary cyproconazole level and FLD, it was reported that accumulation of cyproconazole could also produce liver toxicity and cause hepatocellular adenomas and carcinomas in rodents [48, 49]. Thus, the impact of cyproconazole on FLD could not be neglected.

Our results also indicated that chlorpyrifos was a risk factor for FLD and it was correlated with previous study [36] that chlorpyrifos inhibits diet-induced thermogenesis in BAT and this important factor contributing to the development of obesity and insulin resistance. However, because of the high degree of discrimination of urinary paraquat level, paraquat cannot be considered a definite risk factor for FLD. To further explore whether urinary pesticides level can predict FLD and then provide a basis for the development of new diagnostic kits for clinical practice, ROC analysis was performed. Unexpectedly, the AUC of chlorpyrifos was 0.924 which indicated that chlorpyrifos might serve as independent predictors of FLD.

As mentioned before, the accumulation level of environmental pollutants in the body detecting by exposomes could dynamically reflect health status. Although it represents a cross-sectional study, our study innovatively combines the exposomes and medical imaging diagnosis. MRI has certain advantages in terms of diagnosing FLD and also combined with other omics. However, the dynamic change and convenient detection provide by exposomes would better lead to insights of health. The higher accumulation level of chlorpyrifos and other environmental pollutants in people with high BMI definitely will undoubtedly increase their awareness of health and will encourage them to reduce their exposure to related toxins. Then it would kill two birds with one stone which reducing the level of environmental pollutants and alleviating the severity of the associated disease.

Overall, this is the first investigation of urinary chlorpyrifos, paraquat, and cyproconazole levels in people with FLD. Urinary levels of chlorpyrifos and paraquat were increased in the people with FLD and were positively correlated with the severity of FLD. Moreover, the development of diagnosis kits targeting urinary pesticides could provide new ideas for the diagnosis and prevention of FLD. However, the size of samples included is not enough, so the results of this paper have certain limitations. It is necessary to further expand the sample size in the follow-up studies and subgroup analysis is also needed to explore the relationship between urinary pesticides level and FLD. Beyond that, in the future work, we also need to add the validation part to further prove the efficiency of targeted detection for environmental chemicals.

## Conclusion

The present findings indicate urinary chlorpyrifos and paraquat were positively correlated with the severity of fatty liver. Moreover, urinary chlorpyrifos and paraquat have the potential to be considered as the predictors for development of FLD. Thus, this study may provide a new perspective from the environmental factors for the diagnosis, prevention, and treatment of FLD.

## Data Availability

The datasets used and/or analysed during the current study are available from the corresponding author on reasonable request.

## Abbreviations

MRI: Magnetic resonance imaging
FF: Fat fraction
FLD: Fatty liver disease
LC-MS: Liquid chromatography-mass spectrometry
ROI: Region of interest
