# Publisher Correction: Association of urinary chlorpyrifos, paraquat, and cyproconazole levels with the severity of fatty liver based on MRI

**DOI:** 10.1186/s12889-024-18406-z

**Published:** 2024-03-26

**Authors:** Peiqi Ma, Hongliang Gao, Ning Shen, Lei Zhang, Yang Zhang, Kai Zheng, Boqun Xu, Jian Qin, Jian He, Tao Xu, Yan Li, Jing Wu, Yushan Yuan, Bin Xue

**Affiliations:** 1 Medical imaging center, Fuyang People’s Hospital, 236000 Anhui, Fuyang China; 2 Core Laboratory, Department of Clinical Laboratory Sir Run Run Hospital, Collaborative Innovation Center for Cancer Personalized Medicine, Nanjing Medical University, 211166 Nanjing, China; 3 School of Clinical Medicine, Wannan Medical College, 241000 Wuhu, Wuhu China; 4 China Exposomics Institute (CEI) Precision Medicine Co. Ltd, 200120 Shanghai, China; 5 Jiangsu Engineering Research Center of Stomatological Translational Medicine, Nanjing Medical University, 210029 Nanjing, China; 6 Department of Obstetrics and Gynecology, the Second Affiliated Hospital of Nanjing Medical University, 210011 Nanjing, China; 7 Department of Orthopaedics, Sir Run Run Hospital, Nanjing Medical University, 211100 Nanjing, China; 8 Department of Nuclear Medicine, Nanjing Drum Tower Hospital, Affiliated Hospital of Medical School, Nanjing University, 210029 Nanjing, China; 9 Department of General Surgery, The Affiliated Changzhou Second People?s Hospital of Nanjing Medical University, Nanjing Medical University, 213003 Changzhou, China

**Correction to: BMC Public Health (2024) 24:807**

https://doi.org/10.1186/s12889-024-18129-1

In the original publication of this article the following statement was omitted:

Peiqi Ma and Hongliang Gao contributed equally to this paper.

The original article has been updated to rectify this error.

